# Deep mutationally scanned CHIKV E3/E2 virus library maps viral amino acid preferences and predicts viral escape mutants of neutralizing CHIKV antibodies

**DOI:** 10.1128/jvi.00081-25

**Published:** 2025-03-27

**Authors:** Megan M. Stumpf, Tonya Brunetti, Bennett J. Davenport, Mary K. McCarthy, Thomas E. Morrison

**Affiliations:** 1 Department of Immunology and Microbiology, University of Colorado Anschutz Medical Campus 129263, Aurora, Colorado, USA; Loyola University Chicago - Health Sciences Campus, Maywood, Illinois, USA

**Keywords:** viral immunity, alphavirus, monoclonal antibodies

## Abstract

As outbreaks of chikungunya virus (CHIKV), a mosquito-borne alphavirus, continue to present public health challenges, additional research is needed to generate protective and safe vaccines and effective therapeutics. Prior research established a role for antibodies in mediating protection against CHIKV infection, and the early appearance of CHIKV-specific IgG or IgG neutralizing antibodies protects against progression to chronic CHIKV disease in humans. However, the importance of epitope specificity for these protective antibodies and how skewed responses contribute to the development of acute and chronic CHIKV-associated joint disease remains poorly understood. Here, we describe the deep mutational scanning of one of the dominant targets of neutralizing antibodies during CHIKV infection, the E3/E2 (also known as p62) glycoprotein complex, to simultaneously test thousands of p62 mutants against selective pressures of interest in a high throughput manner. Characterization of the virus library revealed achievement of high diversity while also selecting out nonfunctional virus variants. Furthermore, this study provides evidence that this virus library system can comprehensively map sites critical for the neutralization function of antibodies of both known and unknown p62 domain specificities.

**IMPORTANCE:**

Chikungunya virus (CHIKV) is a mosquito-borne alphavirus of global health concern that causes debilitating acute and chronic joint disease. Prior studies established a critical role for antibodies in protection against CHIKV infection. Here, we describe the generation of a high-throughput, functional virus library capable of identifying critical functional sites for anti-viral antibodies. This new tool can be used to better understand antibody responses associated with distinct CHIKV infection outcomes and could contribute to the development of efficacious vaccines and antibody-based therapeutics.

## INTRODUCTION

Chikungunya virus (CHIKV), a mosquito-borne alphavirus, causes severe acute and chronic joint pain and inflammation, and is a public health threat (1–5). Moreover, CHIKV infection can be fatal; the case-fatality ratio has been estimated between 1 and 2.2 deaths per 1,000 cases (6, 7). Prior research established a role for anti-CHIKV antibodies (Abs) in control of CHIKV infection, and human studies suggest that the early appearance of CHIKV-specific IgG or IgG neutralizing Abs (nAbs) protects against progression to chronic CHIKV disease (8–14). However, the importance of epitope specificity of protective Abs and how skewed responses contribute to the resolution of symptoms or progression to chronic disease remain poorly understood.

CHIKV is an enveloped, positive-sense RNA virus with an ~12 kb genome that encodes two open reading frames (ORFs) (15, 16). The first ORF encodes a nonstructural (ns) polyprotein that is processed to produce four ns proteins (nsP1-4) that mediate RNA synthesis. The second ORF encodes the viral structural proteins capsid-p62-6K/TF-E1. E1 and p62 co-translationally associate within the ER and are glycosylated (17). Within the secretory pathway, p62 is cleaved by furin into the mature E2 and E3 glycoproteins (18). For some alphaviruses, including CHIKV, E3 can remain bound to the mature E2–E1 heterodimer (19, 20). E2 and E1 heterodimers form 80 trimeric spikes on the virion surface. The E2 protein is comprised of three domains (A, B, and C), with domain A positioned toward the center of the spike, domain B on the tip of the spike, and domain C next to the viral membrane. E1 is a class II fusion protein with three domains (I, II, and III). E2 and E1 are the dominant targets for nAbs (21–27).

Here, we deep mutationally scanned (DMS) (28) CHIKV p62 to simultaneously test thousands of p62 mutants against selective pressures of interest in a high throughput manner. DMS of viral proteins has been employed for several viruses, including HIV, SARS-CoV-2, hepatitis C virus, Mayaro virus, Zika virus, and others (28–36). This method has a variety of applications, ranging from the evaluation of therapeutic monoclonal Abs (mAbs) and their epitopes to informing viral evolution models (28, 37–41). Certain DMS methods involve the use of replication-incompetent systems (34, 35, 42, 43). Because CHIKV p62 (E2/E3) is involved in cell entry and egress, we generated a full-length CHIKV plasmid system to elucidate how viral fitness may be affected by certain mutations.

In this study, we evaluated the diversity of the CHIKV-p62-DMS library and inferred the mutational tolerance of CHIKV p62. We then utilized two well-characterized CHIKV mAbs (CHK-152 and CHK-265) (21–26) to verify our library’s capacity to identify important functional sites for nAbs. In addition, we tested the library’s capacity to map sites that influence the neutralizing activity of a mAb whose precise target is undefined (CHK-11) (21). Results from our characterization of the CHIKV-p62-DMS virus library revealed high diversity while also selecting out nonfunctional variants. Furthermore, our data provide evidence that this system can comprehensively identify sites that alter neutralization by mAbs of both known and unknown p62 domain specificities.

## RESULTS

### Characterization of the CHIKV-p62-DMS virus library reveals high diversity and functional variant selection

**Fig 1:**
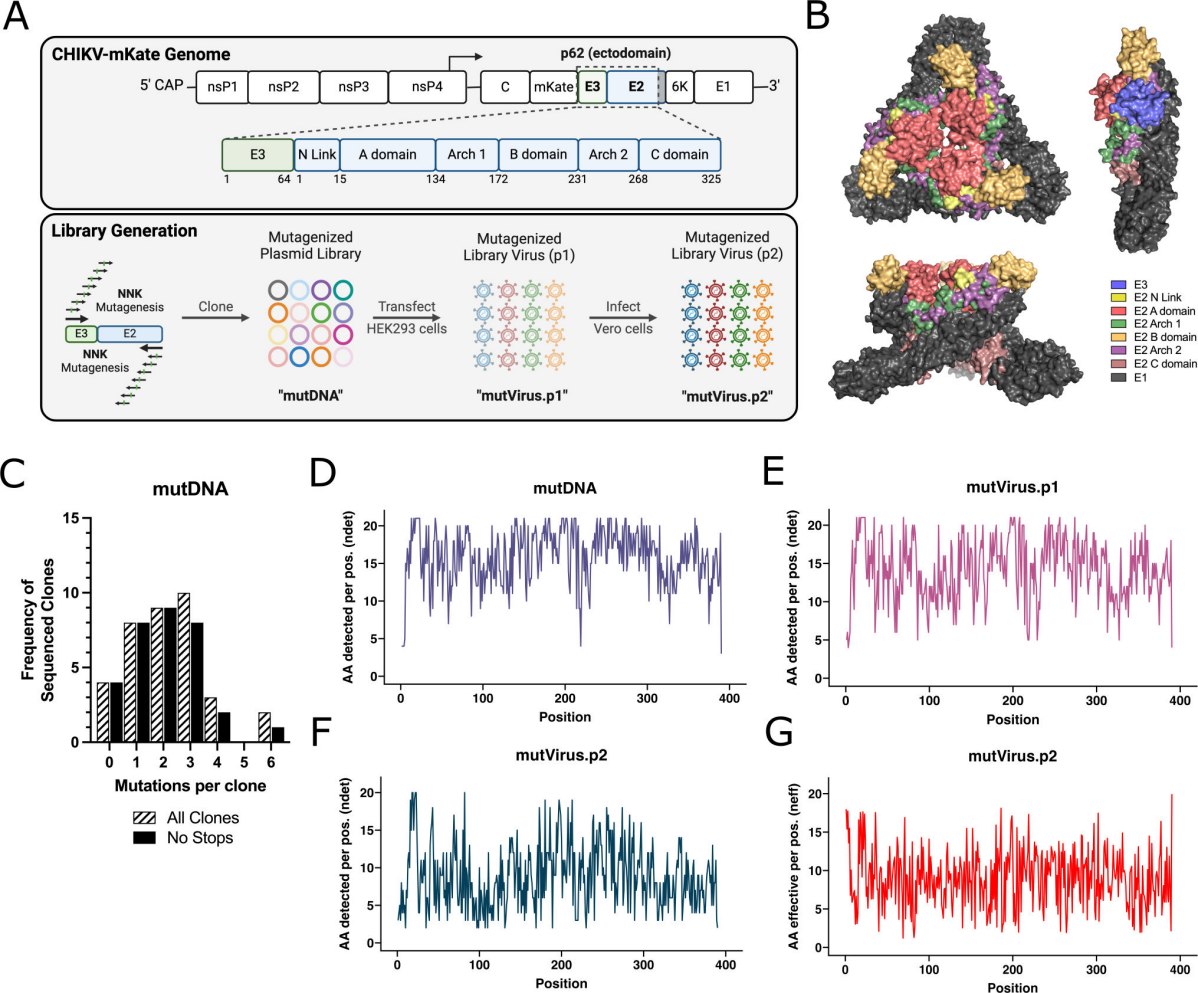
Generation of a deep mutationally scanned CHIKV p62 full-length virus library. (A) Schematic of the CHIKV genome (box indicates mutagenized region), procedure for generation of the mutagenized libraries, and naming scheme for each of the library iterations. (B) Reference structural models of the CHIKV E2/E1 trimeric glycoprotein (PDB: 3J2W) from top-down (top left) and side view (bottom left) and mature CHIKV p62/E1 glycoprotein complex (PDB: 3N42) from the side view (right) with individually colored domains (E3: light blue, E2 N Link: light yellow, E2 A domain: light red, E2 Arch 1: light green, E2 B domain: light orange, E2 Arch 2: light purple, E2 C domain: light brown, E1: gray). (C) Sanger sequencing results for individual plasmid DNA clones. The lined black bars represent the number of nonsynonymous mutations per full-length CHIKV p62 clone (Sanger primers available in Materials and Methods). The solid black bars exclude any sequences containing stop codons. The average number of mutations per clone for the lined and solid black bars are 2.2 and 2.0, respectively. The distribution of these mutations across the CHIKV p62 region is detailed in Fig. S1E. (D–F) Following deep sequencing, the total number of detected amino acids per codon position (“ndet”), for the (D) mutDNA, (E) mutVirus.p1, and (F) mutVirus.p2 libraries are plotted for the entire mutagenized CHIKV p62 region. Results for the wtDNA sequencing control are shown in Fig. S1F. (G) The amino acid preferences for mutVirus.p2, reported as the number of effective amino acids per codon position (“neff”), are plotted for the entire mutagenized CHIKV p62 region in red.

NAbs against CHIKV target the E2 and E1 surface glycoproteins (21–27), but little is known about the role of E3 in the nAb response. We deep mutationally scanned the p62 ectodomain to identify mutations in E3/E2 that impact Ab-mediated inhibition of CHIKV infection (Fig. 1A and B). The CHIKV-p62-DMS virus library was generated in the context of a pCHIKV-CMV-mKate plasmid (Fig. S1A; see Materials and Methods) (44). The plasmid library (mutDNA) was transfected into HEK293 cells, and virus-containing culture supernatants were collected at 48 hours post-transfection (hpt) to generate the first virus library (mutVirus.p1). To select the virus library for functional variants, Vero cells were inoculated with mutVirus.p1 (MOI of 0.01 FFU/cell) and virus-containing culture supernatants were collected at 48 hours post-infection (hpi) to generate mutVirus.p2 (Fig. 1A). Vero cells were chosen because these cells are commonly used for mAb epitope mapping studies and neutralization assays (21–23, 26), allowing us to compare our findings with prior work. Two replicate libraries were independently generated, and both libraries were mutagenized to similar extents (Fig. S1B through D).

To evaluate mutagenesis efficiency, plasmid DNA isolated from 40 bacterial colonies, out of an estimated 2.1 × 10^4^ total, were Sanger sequenced (Fig. 1C and Fig. S1B). Four of the forty (10%) clones either lacked an insert from the ligation reaction or were contaminants. The average number of nonsynonymous and synonymous mutations in the 36 remaining clones was 2.2 and 0.08, respectively (Fig. 1C and Fig. S1D). Four of the thirty-six clones (11%) were eliminated from the analysis due to the presence of a stop codon, decreasing the average number of nonsynonymous mutations per functional clone to 2.0 (Fig. 1B). The distribution of nonsynonymous mutations showed a relatively even spread among the sampled sequences (Fig. S1E).

To assess library sequence diversity, RNA was isolated from mutVirus.p1 and mutVirus.p2, cDNA was generated, and the mutagenized p62 fragment was PCR-amplified. This same region also was PCR amplified from the mutDNA library and the WT pCHIKV-CMV-mKate plasmid to account for PCR-based and sequencing-based errors, and the resulting amplicons were sequenced. The average number of amino acids (AAs) detected per position (“ndet”) for the mutDNA, mutVirus.p1, and mutVirus.p2 libraries was 15.9, 14.2, and 8.9 AAs, respectively (Fig. 1D through F). In contrast, the wtDNA sample had an average of 2.6 AAs detected per position (Fig. S1F). For mutVirus.p2, we also calculated the standard DMS metric, number of effective AAs per position (“neff”), which was similar with an average of 9.0 AAs (Fig. 1G; see Materials and Methods). These findings suggest that both a high degree of mutagenesis was achieved, and functional selection of the virus library occurred following passaging.

### Elevated diversity in the E2 B domain

To simplify the visualization of the deep sequencing data to the absence or presence of AAs above filters, we developed a new metric, “*ndet”* (see Materials and Methods). This avoids confusion with large-effect sizes on rare mutants in logoplots that did not correspond to the frequency of that mutant in the library. To do this, we developed a software package called *megaLogo* (https://github.com/meganstumpf/megalogo) to visualize this diversity metric. For these logoplots, the height of the AA is inversely proportional to the number of total AAs at the site and does not indicate the relative frequencies of these AAs in the virus library.

**Fig 2:**
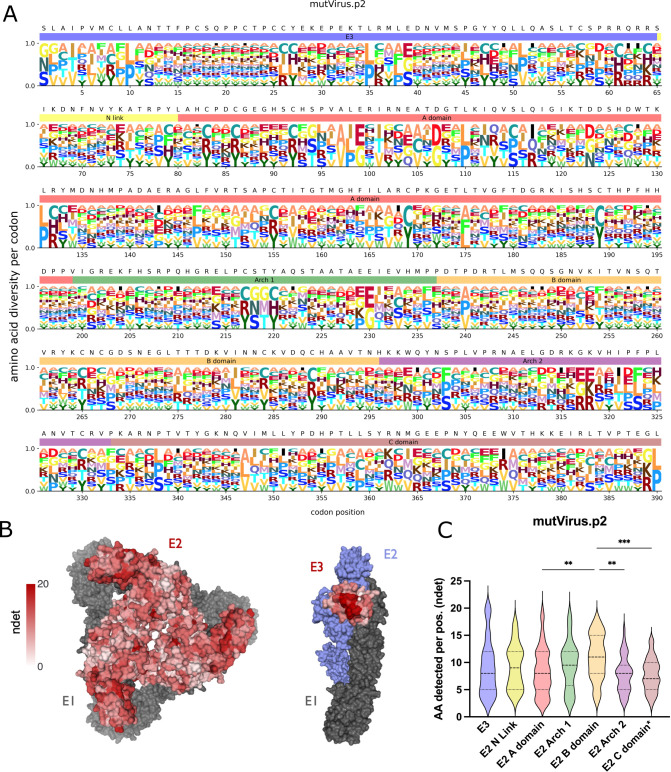
Deep mutational scanning of CHIKV p62 reveals mutational tolerance of the different p62 domains**.** (A) Logoplot showing the diversity of amino acids per codon position for the mutVirus.p2 virus library. WT residues and colored bars for each p62 domain are annotated above each codon position (E3: light blue, E2 N Link: light yellow, E2 A domain: light red, E2 Arch 1: light green, E2 B domain: light orange, E2 Arch 2: light purple, E2 C domain: light brown). Size of each letter is normalized to the number of amino acids detected for that codon position. (B) Using *dms-viz* (46), heatmaps were generated for the trimeric E2/E1 CHIKV envelope glycoproteins cryo-em structure (PDB: 3J2W) and the mature envelope glycoprotein complex (p62/E1; PDB: 3N42). For the trimeric structure, the heatmap represents E2 diversity from a top-down view. For the p62/E1 complex, the heatmap represents E3 diversity from a side view (with E2 colored in blue to highlight E3). E1 is shown in gray. (C) Violin plots showing the relative diversity at each site of each mutagenized domain are plotted. Colors are matched to colors shown in logoplot annotations in panel 2A. *The E2 C domain region only includes the ectodomain portion of the C domain. One-way analysis of variance with Tukey’s test for multiple comparisons. **P < 0.01, ***P < 0.001.

The filtered mutVirus.p2 data set with the *ndet* metric (Fig. 1F) was passed through *megaLogo* and plotted (Fig. 2A). We then generated a heatmap for the corresponding *ndet* values for each residue in the spike (PDB: 3J2W) as well as the p62/E1 complex (PDB: 3N42) to visualize the mutational tolerance of E3 (Fig. 2B). The logoplot and structural heatmaps for mutVirus.p2 (Fig. 2A and B) revealed several individual sites (e.g., WT cysteine residues shown in Fig. 2A) and conformationally relevant regions (e.g., the trimer core and center ridge within the E2 B domain shown in Fig. 2B) where limited mutational tolerance was observed. This is in contrast to more mutationally tolerant spans, such as within E3 (e.g., positions 13–25), and within both the E2 A and B domains, evidenced by larger stacks of residues (Fig. 2A) and many residues with darker red shading (Fig. 2B). Plotting the distribution of *ndet* per site for each domain revealed an increased number of sites in the E2 B domain with higher numbers of detected AAs when compared with other E2 domains (Fig. 2C), suggesting the B domain has more plasticity (45).

### Mapping of Ab escape for CHIKV nAbs identifies new sites of escape

To validate the use of the CHIKV-p62-DMS library virus to identify mechanisms of viral escape from nAbs, we performed escape mutant selection assays with two well-characterized anti-CHIKV mAbs, CHK-152, and CHK-265, as well as an unmapped anti-CHIKV mAb, CHK-11 (21–26). Each mAb was pre-incubated at 37°C for 1 h with either wtVirus or mutVirus.p2 (MOI = 1 FFU/cell) with 2× EC_97_ mAb and virus-mAb mixtures were inoculated onto Vero cells, and the resulting virus output at 24 hpi was sequenced for differential selection. Technical duplicates for all wells were performed and sequenced with each mAb/virus replicate compared to each virus-only replicate, and the final average score was calculated from all comparisons. These results showed modest positive site escape scores throughout p62 for all mAbs; however, individual mutants at several sites were found with high differential selection scores (>10 log_2_) (Fig. S2A and B).

**Fig 3:**
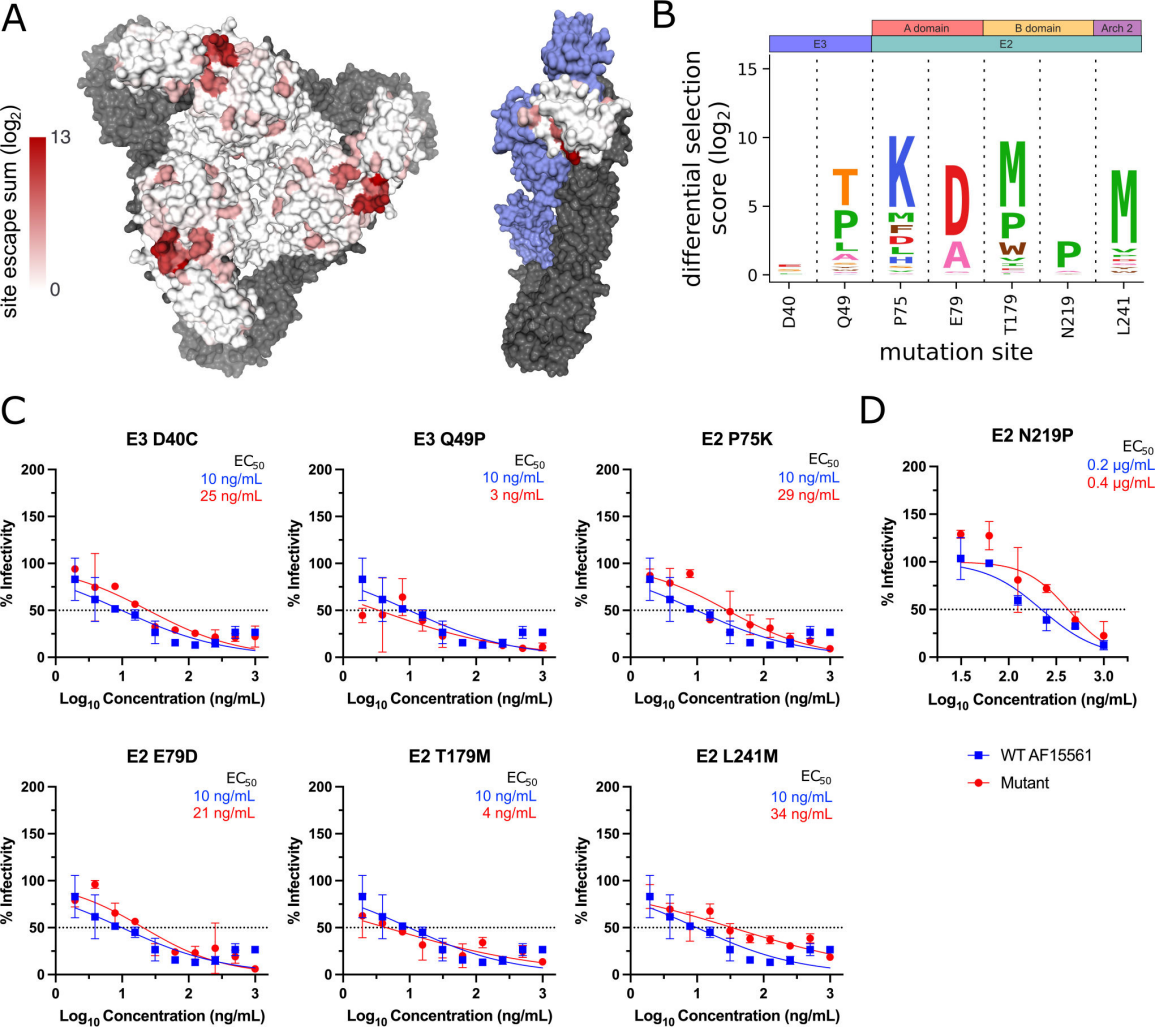
Escape mutant profile for CHK-152 monoclonal antibody reveals modest escape from the selected panel of positive selection mutants. (A) Total site positive differential selection scores for CHK-152 were plotted via heatmap on the trimeric E2/E1 CHIKV envelope glycoproteins (PDB: 3J2W) and the mature envelope glycoprotein complex (p62/E1; PDB: 3N42). For the trimeric structure, the heatmap represents E2 positive site selection from a top-down view. For the p62/E1 complex, the heatmap represents E3 positive site selection from a side view (with E2 colored in blue to highlight E3). E1 is shown in gray for both structures. (B) Sites selected for validation of their sensitivity to neutralization by CHK-152. (C) Focus reduction neutralization test (FRNT) curves for CHK-152 against WT CHIKV and the indicated mutant virus. The dotted line represents the FRNT_50_ threshold. (D) Plaque reduction neutralization test (PRNT) curve for CHK-152 against WT and E2 N219P CHIKV. The dotted line represents the PRNT_50_ threshold.

**Fig 4:**
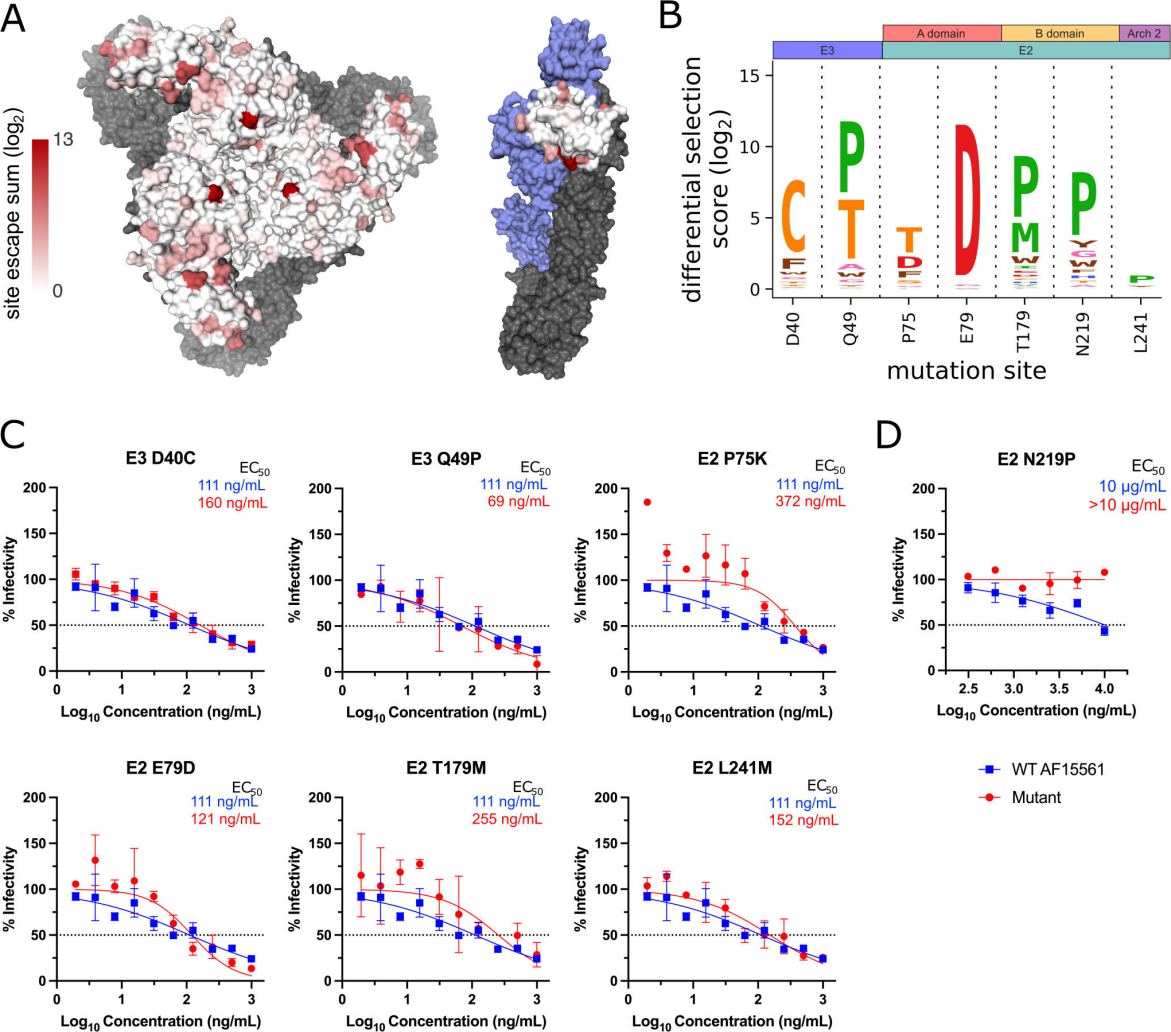
Escape mutant profile for CHK-265 monoclonal antibody reveals modest escape from the selected panel of positive selection mutants. (A) Total site positive differential selection scores for CHK-265 were plotted via heatmap on the trimeric E2/E1 CHIKV envelope glycoproteins (PDB: 3J2W) and the mature envelope glycoprotein complex (p62/E1; PDB: 3N42). For the trimeric structure, the heatmap represents E2 positive site selection from a top-down view. For the p62/E1 complex, the heatmap represents E3 positive site selection from a side view (with E2 colored in blue to highlight E3). E1 is shown in gray for both structures. (B) Sites selected for validation of their sensitivity to neutralization by CHK-265. (C) FRNT curves for CHK-265 against WT CHIKV and the indicated mutant virus. The dotted line represents the FRNT_50_ threshold. (D) PRNT curve for CHK-265 against WT and E2 N219P CHIKV. The dotted line represents the PRNT_50_ threshold.

**Fig 5:**
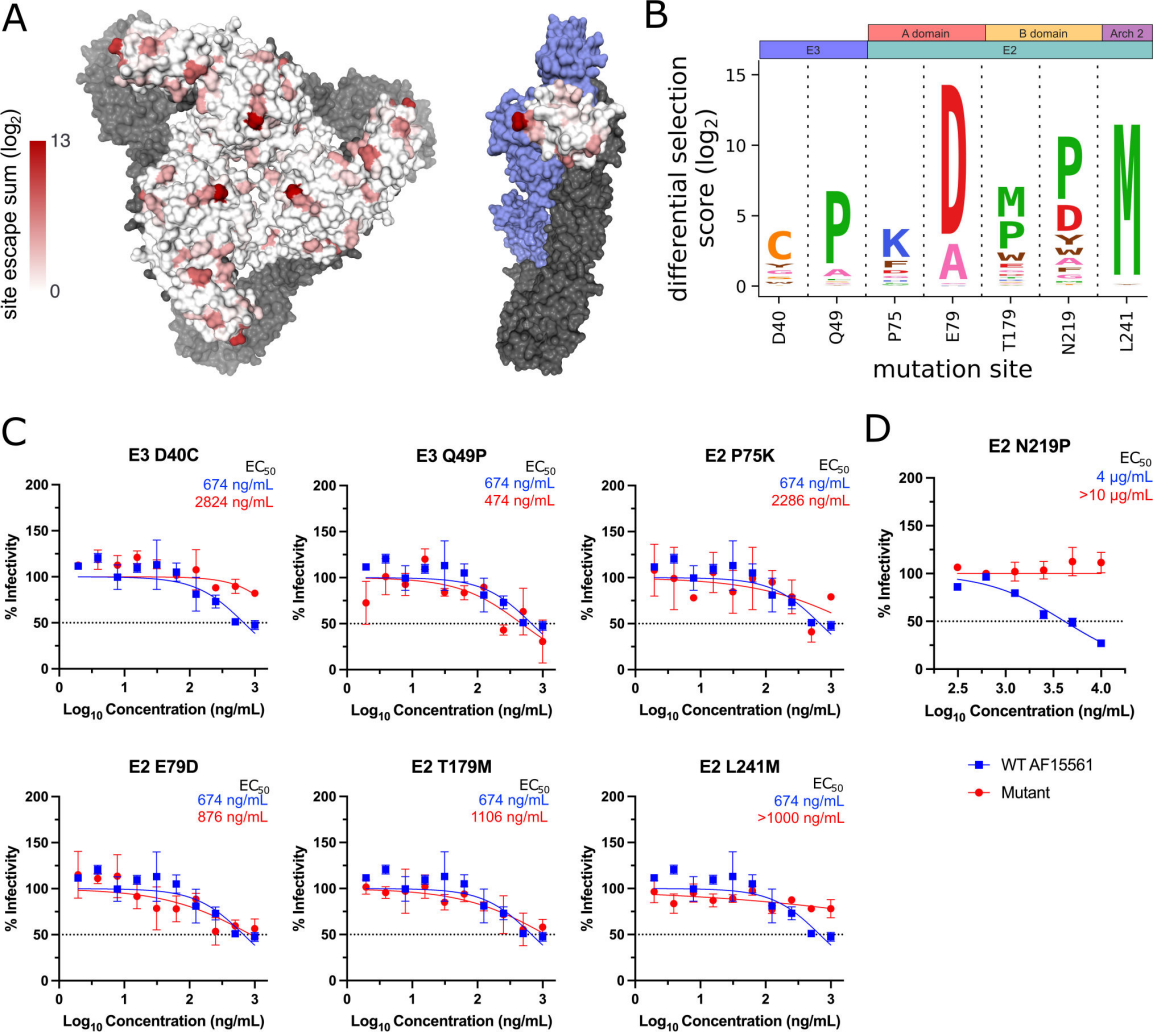
Escape mutant profile for CHK-11 monoclonal antibody reveals modest escape from the selected panel of positive selection mutants. (A) Total site positive differential selection scores for CHK-11 were plotted via heatmap on the trimeric E2/E1 CHIKV envelope glycoproteins (PDB: 3J2W) and the mature envelope glycoprotein complex (p62/E1; PDB: 3N42). For the trimeric structure, the heatmap represents E2 positive site selection from a top-down view. For the p62/E1 complex, the heatmap represents E3 positive site selection from a side view (with E2 colored in blue to highlight E3). E1 is shown in gray for both structures. (B) Sites selected for validation of their sensitivity to neutralization by CHK-11. (C) FRNT curves for CHK-11 against WT CHIKV and the indicated mutant virus. The dotted line represents the FRNT_50_ threshold. (D) PRNT curve for CHK-11 against WT and E2 N219P CHIKV. The dotted line represents the PRNT_50_ threshold.

To validate our findings, we evaluated the extent to which positive escape sites were identified at known contact sites or critical residues for the previously studied mAbs CHK-152 and CHK-265 (Tables S1 and S2) (21–26). Defined as having an average (log_2_) positive differential selection score ≥0.1 across all comparisons, 9/9 (100%) of prior published sites had at least one escape mutant for CHK-152 and 54/59 (92%) for CHK-265 (Fig. S3), indicating consistency with prior studies. Total positive differential selection scores (log_2_) for each site were plotted as a heatmap on the CHIKV spike and the E3-focused p62/E1 heterodimer for CHK-152 (Fig. 3A), CHK-265 (Fig. 4A), and CHK-11 (Fig. 5A).

To determine if the CHIKV-p62-DMS library virus could be used to identify novel sites critical for Ab-mediated inhibition, we selected a panel of mutants at sites outside the known residues described in Tables S1 and S2 to test for neutralization escape (Fig. 3 to 5). In addition, we also tested the extent to which the CHIKV-p62-DMS library virus could be used to map escape from uncharacterized mAbs such as CHK-11. The criteria for selection of these sites included (i) be a novel site within a domain containing known contacts OR be a site within a domain with no known contacts, (ii) score as a top escape mutant for at least two of the three mAbs, (iii) not a previously identified site of escape or contact, and (iv) preferably surface exposed. These criteria produced the following mutants: E3 D40C and E3 Q49P, E2 P75K and E2 E79D (A domain), E2 T179M and E2 N219P (B domain), and E2 L241M (Arch 2) and the respective positive site escape logoplots (generated via *dmslogo*) are shown for each mAb (Fig. 3B, 4B, and 5B).

We produced the panel of individual mutants in CHIKV AF15561 and evaluated neutralization capacity for each mAb. One mutant virus, E2 N219P, was undetectable by our focus formation assay, which relies on CHK-11 as the detection Ab. For this reason, E2 N219P neutralization was evaluated by a plaque reduction neutralization test (PRNT) (Fig. 3D, 4D, and 5D) while all other mutants were evaluated by a focus reduction neutralization test (FRNT) (24). For most mutants, only modest escape was observed against each mAb with a twofold to fourfold change in EC_50_ (ng/mL) (Fig. 3C, 4C, and 5C), except for E2 L241M, which we were unable to calculate an EC_50_ for CHK-11 at the dilutions tested (Fig. 5C). Conversely, the E2 N219P mutation mediated escape from mAbs CHK-265 and CHK-11 (Fig. 4D and 5D) resulting in an inability to calculate an EC_50_ (µg/mL) for both mAbs. In contrast, E2 N219P mediated only modest twofold escape from CHK-152 (Fig. 3D).

### CHK-11 targets an overlapping epitope with CHK-265 and mediates broadly neutralizing activity

**Fig 6:**
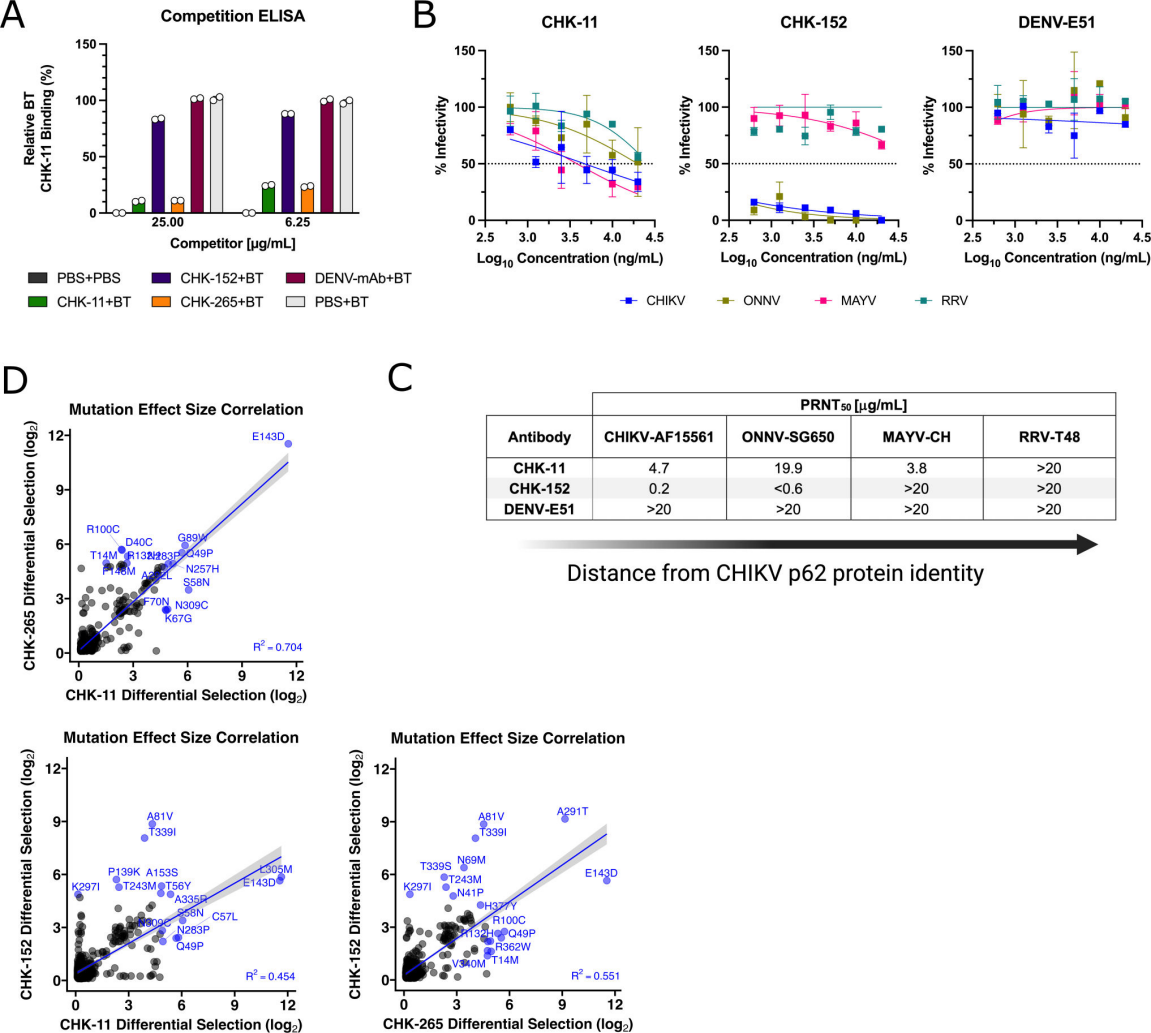
Validation of CHK-11 and CHK-265 similarity. (A) Competition ELISA against CHIKV virions (strain 181/25) with competitor antibodies added prior to the addition of biotinylated CHK-11 (“BT”). Percent binding (absorbance @ 450 nm) relative to PBS+BT-CHK-11 (no competitor; shown in light gray) was determined. (B) PRNT curves against CHIKV, o’nyong’nyong virus (ONNV), Mayaro virus (MAYV) and Ross River virus (RRV) on Vero cells. DENV E51 was included as an isotype, neutralizing antibody negative control. The dotted line represents the PRNT_50_ threshold. (C) Calculation of the PRNT_50_ in µg/mL for each virus-antibody condition, ordered by relatedness to CHIKV. (D) Correlation of shared positively selected mutants between CHK-11, CHK-152, and CHK-265 was evaluated to compare mutation effect sizes (log_2_ differential selection score) using linear regression in R v4.2.1. Top ten mutants for each mAb pair are colored blue.

Due to the observed similarities between the escape profiles for CHK-11 and CHK-265, we evaluated whether CHK-11 and CHK-265 target overlapping epitopes by competition enzyme-linked immunosorbent assay (ELISA) (Fig. 6A). Using an established virion-based ELISA (24), biotinylated CHK-11 (BT CHK-11) was competed with unlabeled competitor Ab. Relative to BT CHK-11 binding with no competitor Ab, CHK-265 blocked binding of CHK-11 to a similar level as the self-competition control (CHK-11+BT CHK-11: 10.5%, CHK-265+BT CHK-11: 11%), suggesting significant epitope overlap or steric hindrance by CHK-265. CHK-152 only moderately reduced binding of CHK-11 (83.5%), suggesting more limited epitope overlap or steric hindrance (Fig. 6A).

Prior studies demonstrated that CHK-265 is a broadly neutralizing mAb against arthritogenic alphaviruses (23). Thus, we investigated if CHK-11 also shared this feature (21–26) by comparing the neutralizing activity of CHK-11 and CHK-152 against three additional arthritogenic alphaviruses, o’nyong’nyong virus (ONNV), Mayaro virus (MAYV), and Ross River virus (RRV) (Fig. 6B and C). Similar to prior data published for CHK-265 (23, 26, 47), CHK-11 neutralized both CHIKV and MAYV, while neutralizing ONNV and RRV to a lesser degree. In contrast, CHK-152 neutralized both CHIKV and ONNV, while only minimal reductions in infectivity were noted for RRV and MAYV (Fig. 6B and C).

Finally, to test if these observed similarities extended back to the escape mutants identified for CHK-11 and CHK-265, we performed a correlation analysis of the differential selection scores (log_2_) for escape mutants shared by both mAbs (Fig. 6D). Using a linear regression model analysis of all positive escape mutants identified, we found a strong correlation between the average effect size of escape mutants for CHK-11 and CHK-265 (R^2^ = 0.704, *P* < 0.0001) with the top ten mutants for each mAb colored in blue (*n* = 15, five shared between antibodies). This contrasted with CHK-11 and CHK-152 (R^2^ = 0.454, *P* < 0.0001) and CHK-265 and CHK-152 (R^2^ = 0.551, *P* < 0.0001) which showed weaker correlations on differential selection scores for individual mutants, indicating CHK-11 and CHK-265 may share more contact sites or conformational dependencies than either Ab when evaluated against CHK-152.

## DISCUSSION

It is important to define how the immune system can be modulated to better protect against future CHIKV outbreaks. Here, we describe the generation, characterization, and functional validation of a CHIKV p62 DMS virus library capable of characterizing critical functional sites of anti-viral Abs to address this need.

Using this library, we found that distinct regions of the CHIKV p62 ectodomain display differential mutational tolerance, with the E2 B domain having a higher proportion of sites capable of tolerating more amino acid substitutions, specifically when compared to the E2 A, Arch 2, and C domains. This may be explained by the wide variety of protein interactions the E2 B domain can engage in, including both cell receptor and Ab binding (45), and this plasticity has been shown to permit rapid adaptation to restrictive cell types *in vitro* (48, 49). Conversely, our analysis also revealed regions of the trimer with less lenience for substitutions, including portions of the surface-exposed region of the E2 A domain and trimer “core,” representing potential targets for therapeutics or vaccine immunogens. Additionally, the function of the E3 glycoprotein has not been fully elucidated. Prior studies have linked E3 to the virus maturation process (48); however, our mutational tolerance data should aid the field in identifying additional E3 functions, as well as the domains and residues critical for those functions.

This study introduced a modification to the previously described DMS mutational tolerance metric known as *neff*, or number of effective amino acids (28, 33, 37, 38, 40), to help reduce potential over-estimation of amino acid preference calculations influenced by initially low mutation frequencies. To evaluate the consistency of our new analysis metric, *ndet*, for mutVirus.p2 with that of *neff*, we demonstrated similar results (average *ndet*: 8.9 AA; average *neff*: 9.0 AA). However, we identified an important difference demonstrating the utility of *ndet*; at both the 5′ and 3′ ends of the mutagenized p62 region, minimal filtering (≥100 reads) of high-quality reads (Phred score ≥24) for each codon-position pairing resulted in few detectable amino acids, yet high numbers of effective amino acids for the same positions, providing important insight that certain positions with low initial read counts may have inflated mutational tolerance values.

We sought to verify the library’s capability to identify known functional sites of well-characterized mAbs targeting different epitopes and confirmed that our mutagenized virus library identified previously published sites critical for each mAb, including specific functional escape mutants (i.e., E2 D59N for CHK-152 [21]). We also tested whether our library could supplement the list of previously identified sites for these mAbs. A select panel of mutagenized viruses confirmed a modest escape mutant in E3, in addition to E2 A and B domain mutants. One mutant, E2 N219P, ablated the neutralization activity of CHK-265, which had been previously identified as an *in vivo* escape mutant during RRV infection (E2 T219P) following CHK-265 treatment (50) but not as a hit in alanine mutagenesis scanning assays (23), further demonstrating the utility of more comprehensive, DMS-derived functional site mapping assays.

We also tested the capacity for the library to identify key residues for neutralization function of an unmapped mAb, CHK-11 (21). Using the same panel of mutants for CHK-11 as was used for CHK-152 and CHK-265, CHK-11 demonstrated a strong similarity to CHK-265 in sensitivity to certain mutants within the panel but was more sensitive to E3 D40C as well as the Arch 2 mutant, E2 L241M. A competition ELISA confirmed CHK-265 either overlaps or sterically hinders CHK-11 binding, and we found that CHK-11 shared the same broadly neutralizing activity profile as CHK-265 and other B domain bnAbs (23, 26, 50). This collection of data, along with correlation analyses with known mAbs CHK-152 and CHK-265, provides evidence that the CHIKV-p62-DMS virus library can be used to map critical Ab sites within the p62 ectodomain and can be further explored for its utility in mapping functional residues of polyclonal serum or additional mAbs in future studies.

This study has limitations. With the method used to introduce mutations, acquiring multiple mutations per clone is a possibility, as shown in our Sanger sequencing results. Additionally, the mutagenized region length exceeds a standard read length on most short-read sequencing platforms. Thus, phenotypes that were observed could be confounded by mutations outside our read frames. To combat this, we verified if deep sequencing results accurately predicted escape mutants by individually introducing representative escape mutations and testing neutralization escape. This did confirm the phenotype expected for many of the mutants tested; however, the degree of escape was more modest for some of the mutations than predicted by sequencing. However, especially for the bnAbs tested, CHK-265 and CHK-11, this may be due to the nature of their broadly neutralizing activity, as described in Kikawa et al. (51). Additionally, it is possible alphaviruses have virus-specific factors that contribute to the degree of differential selection observed when compared with prior DMS Ab site mapping studies that identified higher degrees of escape (31, 32). Future studies could incorporate a larger panel of Abs and escape mutants to determine if our findings extend to other anti-CHIKV mAbs or if Abs narrower than CHK-11/CHK-265 and even CHK-152 would have larger site differential selection scores within linear spans of residues, versus very large individual mutant scores throughout the mutagenized region.

Overall, this study demonstrates a method for characterizing alphavirus mutational tolerance and can be used to map critical sites for nAbs in a high-throughput system. Broadly, this research could provide insights into how the adaptive immune response can be modulated to better protect against infection and disease from CHIKV and other emerging alphaviruses.

## MATERIALS AND METHODS

### Plasmid design

The CHIKV AF15561 sequence (GenBank EF452493) was cloned downstream of a human CMV promoter. An SV40 poly A sequence was inserted downstream of the CHIKV 3′-UTR, and the hepatitis delta ribozyme was inserted adjacent to the poly A tail of the viral genome. To enable identification of infected cells and as a biosafety feature (52–54), the viral genome was engineered to encode the fluorescent protein mKate in-frame in the viral structural polyprotein (55). ApaI and XhoI sites were introduced, flanking the p62 ectodomain, to facilitate cloning of mutagenized p62 fragments into the pCHIKV-CMV-mKate backbone. Two variations of this plasmid were produced: (i) digested with ClaI and religated to remove the CHIKV nonstructural proteins and prevent background caused by template carry-over during library mutagenesis, and (ii) two stop codons were engineered into the E3 portion of the mutagenized p62 region to reduce downstream overrepresentation of WT virus.

### Mutagenesis and cloning

The pCHIKV-CMV-mKate plasmid was digested with ClaI and re-ligated to isolate the p62-containing fragment. The resulting plasmid was used to amplify the E3/E2 glycoprotein region for mutagenesis. PCRs were performed as previously described with slight modifications (37, 56). Primers for mutagenesis were designed using the NNK approach and generated using the *Codon Tiling Primers* Python script that prioritizes equal melting temperatures for oligos over equal-length oligos, then pooled all primers at equimolar concentration (31, 57). Forward and reverse mutagenesis PCRs, as well as the joining reaction PCRs, were performed for a single round to reduce codon mutation frequency. The joining reaction PCR product was gel-purified using the Zymoclean DNA Gel Recovery Kit (ZymoResearch). The mutagenized E3/E2 region and the pCHIKV-CMV-mKate plasmid (with two subsequent stop codon mutations in E2) were digested with ApaI and XhoI (NEB). Following dephosphorylation using alkaline phosphatase (NEB), ligation was performed using T4 DNA Ligase (NEB).

The ligation reaction product was electroporated into ElectroMAX DH10B *E. coli* cells (ThermoFisher) to generate the plasmid library. Transformation was performed using 0.1 cm cuvettes (BioRad) at 2.0 kV, 200 W, 25 mF, then incubated in SOC for 1 h at 37°C and plated on to 2× LB-Amp plates and incubated at 37°C for 14–18 h. Individual colonies were selected, grown in 2× LB-Amp broth, and miniprepped for analysis by Sanger sequencing. The remaining plated colonies were scraped and grown in 2× LB-Amp broth overnight. The total number of colonies was estimated by counting plates with either 1:40 or 1:400 dilutions of transformed bacteria.

### Plasmid library transfection and library virus infection

Mutant virus libraries were generated by transfection of HEK293 cells (ATCC: CRL-1573). Cells were plated for transfection with 30 µg mutDNA in 150 mm TC-treated dishes with 1.0 × 10^7^ cells and incubated overnight at 37°C prior to transfection. DNA transfection was performed using Lipofectamine 3000 (ThermoFisher) per manufacturer recommendations. Virus supernatants and cells were collected at 48 hpt.

Library virus infection was performed in 150 mm dishes seeded with 1.0 × 10^7^ Vero cells. Virus (MOI of 0.01 FFU/cell) was adsorbed to cells for 1 h at 37°C. After the adsorption period, D10 media was added, and cells were incubated at 37°C for 48 h.

### Virus titering assays

Focus formation assays (FFA) were performed as previously described (58). Foci were counted using the CTL BioSpot analyzer and software (Cellular Technology). For ONNV, MAYV, RRV, and CHIKV E2 N219P (and WT CHIKV control virus), titer assays were performed using a plaque assay as described previously (58). Data were analyzed using Microsoft Excel and GraphPad Prism v10.1.1.

### Purification of monoclonal antibodies

Hybridomas for CHK-11, CHK-152, and CHK-265 were kindly provided by Michael Diamond (Washington University) (21). In brief, hybridomas were expanded in complete IMDM media (Gibco; IMDM, 10% FBS, 1% penicillin-streptomycin) and transferred to a 2 L roller bottle at a density of 5 × 10^5^ cells/mL with 500 mL of IMDM and incubated in a 37°C roller incubator for 7 days. At 1 week, cells were supplemented with an additional 500 mL of complete IMDM without FBS. Cells were pelleted at 4°C and harvested when most cells were dead (>90%). Supernatant was filter sterilized and concentrated using Centricon Plus-70 100 kDa filters (Millipore Sigma). Concentrated supernatants were purified with the Nab Protein G Spin Kit (Life Technologies) and concentrated again using Amicon Ultra-15 10 kda filters (Millipore Sigma). Final concentrates were quantified via a virion-based ELISA described previously (24).

### Plaque and focus reduction neutralization tests

FRNTs were performed as previously described (21, 24). Prior to the addition of virus to Vero cells, virus was preincubated with a dilution series of mAb for 1 h at 37°C in a 96-well V-bottom plate along with virus-only controls. Following the addition of overlay at 2 hpi, cells were fixed with 2% PFA at 14–16 hpi. Data analysis was performed using Microsoft Excel and GraphPad Prism v10.1.1. The PRNT and plaque formation assay protocol is detailed in Hawman et al., with modification for a dilution series of monoclonal antibodies (as performed in the FRNT assay) instead of serum (with heat inactivation) (10, 24).

### Monoclonal antibody challenge

MAbs were incubated with virus at an MOI of 1 in 100 µL of diluent for 1 h at 37°C, and virus-mAb mixtures were added to Vero cells. Plates were incubated at 37°C for 1 h, cells were washed five times with 1× PBS, and 1 mL of fresh medium was added. At 16–18 hpi, supernatants were harvested and stored at −80°C. Viral titers and viral RNA were harvested and sequenced as described above.

### Mutant virus production

Site-directed mutagenesis (SDM) of plasmid DNA was performed using the QuikChange II XL SDM Kit (Agilent Technologies) according to the manufacturer’s instructions, transformed into XL-10 Gold cells, miniprepped, and then sequence confirmed. Select clones were midiprepped (ZymoResearch) and prepared for *in vitro* transcription.

*In vitro* transcription was performed as previously described (58). In brief, plasmids were linearized with NotI (NEB). *In vitro* transcription was performed using mMessage mMachine Kit (Invitrogen) per manufacturer instructions. BHK-21 cells (ATCC: CCL-10) were electroporated as described (59) and harvested 24 h later. Viral titers were determined by FFA.

### Verification of critical sites for CHK-152 and CHK-265

Literature review results for critical sites previously identified for neutralizing antibody CHK-152. Given all sites have been identified in E2, they are detailed in Tables S1 and S2, E2 position numbering was included along with the respective p62 numbering for referencing any results described within this manuscript. If the strain in the study differed from CHIKV AF15561, that was noted. The origin strain residue for that site was also included, and if the amino acid differed in the AF15561 strain, the AF15561 residue was denoted as (X). In the case the study identified a mutation that escaped the tested function, this was recorded. All identified escape mutants in our study were listed in the “Library Escape Mutants” column in order of largest (log_2_) positive differential selection score to least (log_2_) positive differential selection score. The method the study used to identify the critical residue was also listed and referenced. Mutants with average (log_2_) positive differential selection scores ≥0.1 across all comparisons were included and ranked based on their average score and bolded if identified as an escape mutant in the referenced studies.

### RNA isolation, RT-PCR, and amplicon PCR for library preparation

RNA isolation of viral samples and RT-PCR for generation of cDNA was performed as described previously (60). To improve sequencing coverage of the mutagenized p62 region, an amplicon PCR was performed using KOD Hot Start (Millipore Sigma) as previously published (56) utilizing the amplifying primers described below (see section Primers). Amplicons were purified using the QIAquick PCR Purification Kit (Qiagen) and submitted for mechanical shearing (Covaris) and library preparation to the University of Colorado Genomics Shared Resource Facility (RRID: SCR_021984). Library preparation was performed using the Ovation Ultralow System V2 kit (Tecan).

### Sequence analysis

Samples were sequenced with 2 × 150 bp reads at a minimum depth of 25M/50M PE reads on the Illumina NovaSeq 6000. Raw fastq reads were evaluated for sequencing quality using *FastQC* software v0.11.9. Reads were trimmed and cleaned using *cutadapt* software v3.4. The quality of trimmed reads was re-evaluated for quality using *FastQC*. These reads were subsampled with *seqtk* to 25M PE reads and aligned to the WT pCHIKV-CMV-mKate plasmid reference genome (see “Data availability”) and codons called using *VirVarSeq* software (61). The *VirVarSeq* output was filtered for mutations with a minimum average Phred quality score of 24 or higher and analyzed using a custom code in Python v3.9.2 and R v4.2.1. (see “Data availability”). Amino acid preference (“neff”) and differential selection (“mutdiffsel”) scores were calculated using *dms_tools2* (57). Structures were pulled from the Protein Data Bank (PDB: 3N42, 3J2W) and heatmap projections were generated using *dms-viz* (46). Reference structures with color-coded domains (Fig. 1B) were generated using PyMOL v3.1.3.1. Bar plots, violin plots, and neutralization curves were plotted and analyzed using GraphPad Prism v10.1.1. Miniprep clone sequences were aligned to the pCHIKV-CMV-mKate reference genome using Geneious Prime v2022.1.1.

To calculate the *ndet* metric, we first filtered the *VirVarSeq* output codon matrices with the following conditions: (i) greater than 100 counts for that codon-position pair, and (ii) an average forward and reverse read minimum Phred quality score ≥24. Then, the remaining unique residues were summed for each position (*ndet*). For plotting the filtered residues into logoplots, each position stack was set to total a value of 1.0, and the height of the AA (*F*_*site*_) is inversely proportional to the number of total AAs at the site (1/*ndet*). Thus, the size of each residue does not indicate the relative frequencies of these AAs in the virus library but rather the mutational tolerance at that codon position;


(1) $${\text{F}}_{\text{site}}=\frac{1}{\text{ndet}}$$

Calculation of the mutation differential selection metric (*mutdiffsel*) was performed as described previously (57). In brief, the differential selection score ($s_{r,x}$) at each site *r* for each mutation *x* in each selection versus mock-selection controls is calculated where $E_{r,x}$ represents the relative enrichment of observed counts of each codon over the WT codon count, divided by the relative enrichment in the mock-selection condition;


(2) $${\text{s}}_{\text{r,x}}=\log_{2}{\text{E}}_{\text{r,x}}$$

### Primers

PCR primers used for both amplification and joining reactions: FwdPrimer: 5′-AGACGTTGAGTCCAACCCTGGGCCCA-3 and RevPrimer: 5′-CTCGTTGTTGCCCCACGTGACCTCGAG-3′. The NNK degenerative primers used for mutagenesis are available at: https://github.com/tmorrisonlab/chikvdms-mAb-paper. Primers for Sanger sequencing of miniprep clones CHIKV_Lib_FwdSeq1: 5′-CAAGGAGGCCGACAAAGAGAC-3′, CHIKV_Lib_FwdSeq3: 5′-CCAGGTTTCCTTGCAAATCGG-3′, CHIKV_Lib_RevSeq1: 5′-GCTAGGTACGGTCTTGTGGC-3′, CHIKV_Lib_RevSeq2: 5′-CCACCGTCAGAGTTTCTCC-3′, CHIKV_Lib_RevSeq5: 5′-CAGGAGTACGAACGAGGCC-3′.

## Data Availability

The deep sequencing data files and detailed code are available on Zenodo under the following DOIs: 10.5281/zenodo.14269994, 10.5281/zenodo.14510616, and 10.5281/zenodo.14510626. All code, inputs, and any additional outputs not published in this paper, including reference sequence files, are located at: https://github.com/tmorrisonlab/chikvdms-mAb-paper.

## SUPPLEMENTAL MATERIAL

Supplemental material jvi.00081-25-s0001.pdf Figures S1 to S3; Tables S1 and S2.

ASM does not own the copyrights to Supplemental Material that may be linked to, or accessed through, an article. The authors have granted ASM a non-exclusive, world-wide license to publish the Supplemental Material files. Please contact the corresponding author directly for reuse.
