# Supplementary material for: Deep mutationally scanned CHIKV E3/E2 virus library maps viral amino acid preferences and predicts viral escape mutants of neutralizing CHIKV antibodies
Source: J Virol. 2025 Mar 27;99(4):e00081-25. doi: 10.1128/jvi.00081-25 (PMC11998513; doi:10.1128/jvi.00081-25)
Supplement: Supplemental material — Figures S1 to S3; Tables S1 and S2. [file jvi.00081-25-s0001.pdf]

SUPPLEMENTARY MATERIALS

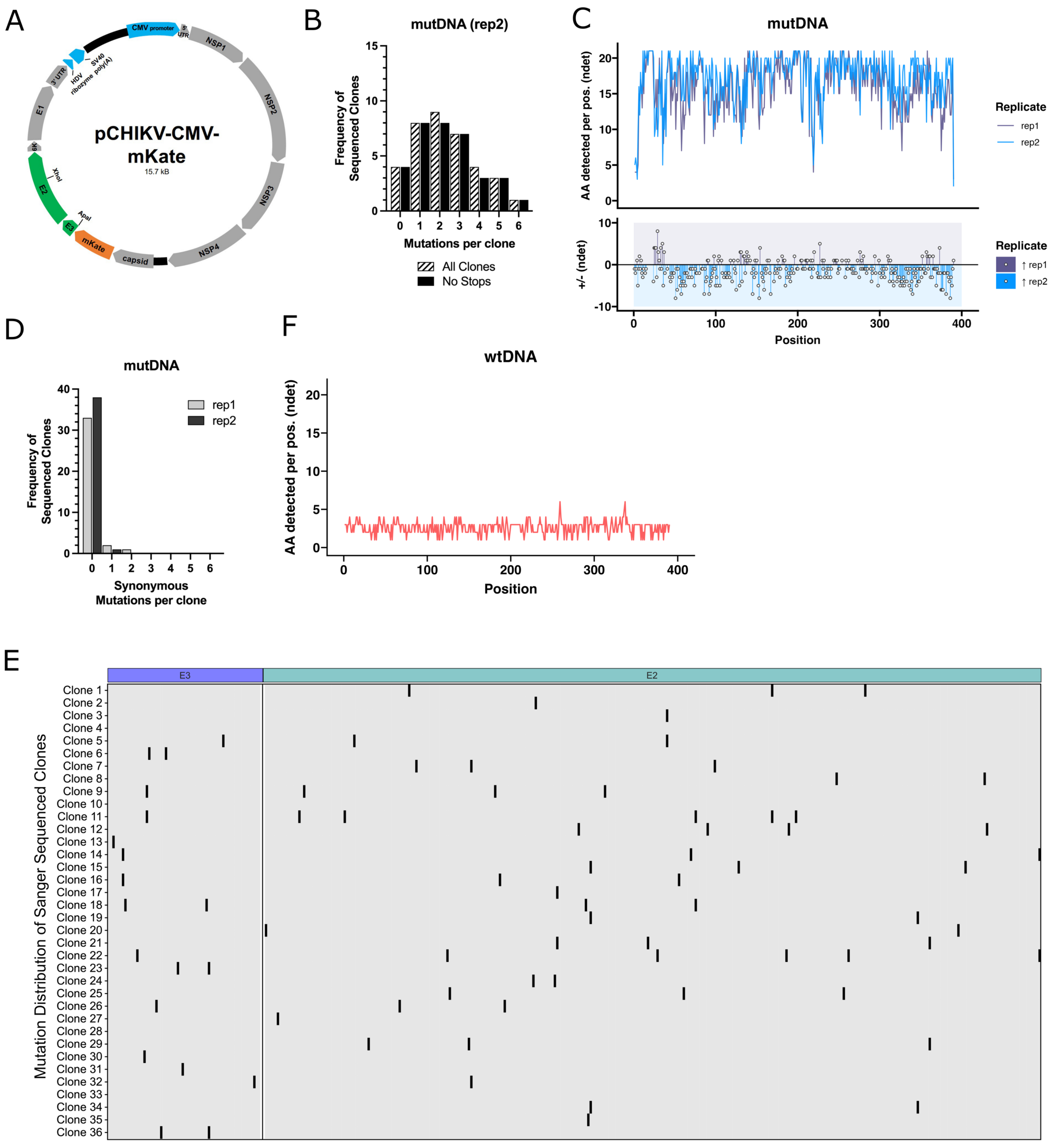

Figure S1. Distribution of clonal mutations across representative mutDNA minipreps and diversity in wtDNA sequencing control. (**A**) Plasmid design for pCHIKV-CMV-mKate. (**B**) Sanger sequencing results for individual plasmid DNA clones for an independently-generated replicate (2 of 2) of the CHIKV-p62-DMS mutDNA library. The lined black bars represent the number of nonsynonymous mutations per full-length CHIKV p62 clone (Sanger primers available in **Materials and Methods**). The solid black bars exclude any sequences containing stop codons. (**C**) Mutagenesis efficiency measured by *ndet* for both replicates of the mutDNA plasmid library.

The top panel represents lineplots of the *ndet* per position for replicate 1 (“rep1”; purple) and replicate 2 (“rep2”; light blue) showing similarity in library diversity. The bottom panel represents the residuals for both libraries compared with each other, where sites with increased amino acids (AAs) in rep1 are plotted as positive (purple region) and sites showing increased diversity in rep2 are plotted as negative (light blue region). (**D**) Sangar sequencing results for individual plasmid clones and number of synonymous mutations per full-length CHIKV p62 clone (Sangar primers available in **Materials and Methods)**. The mutDNA replicate 1 per-clone mutation frequency results are shown in light gray, and results for replicate 2 are shown in dark gray. (**E**) Forty plasmid clones were Sangar sequenced and aligned to the pCHIKV-CMV-mKate WT control for number of amino acid mutations in the entire mutagenized p62 region. E3 is annotated by the periwinkle box on the left, E2 in teal on the right. Each black line represents a nonsynonymous mutation from the WT sequence. Four of the 40 clones (10%) were excluded from the alignment because they either represented vector-only clones or were non-CHIKV plasmid contaminants. Alignment performed using Geneious and graphed with GraphPad Prism. (**F**) The total number of amino acids detected per codon position (‘ndet’) for the wtDNA sequencing control. For all differential selection analyses, the wtDNA control is subtracted from all experimental conditions.

A

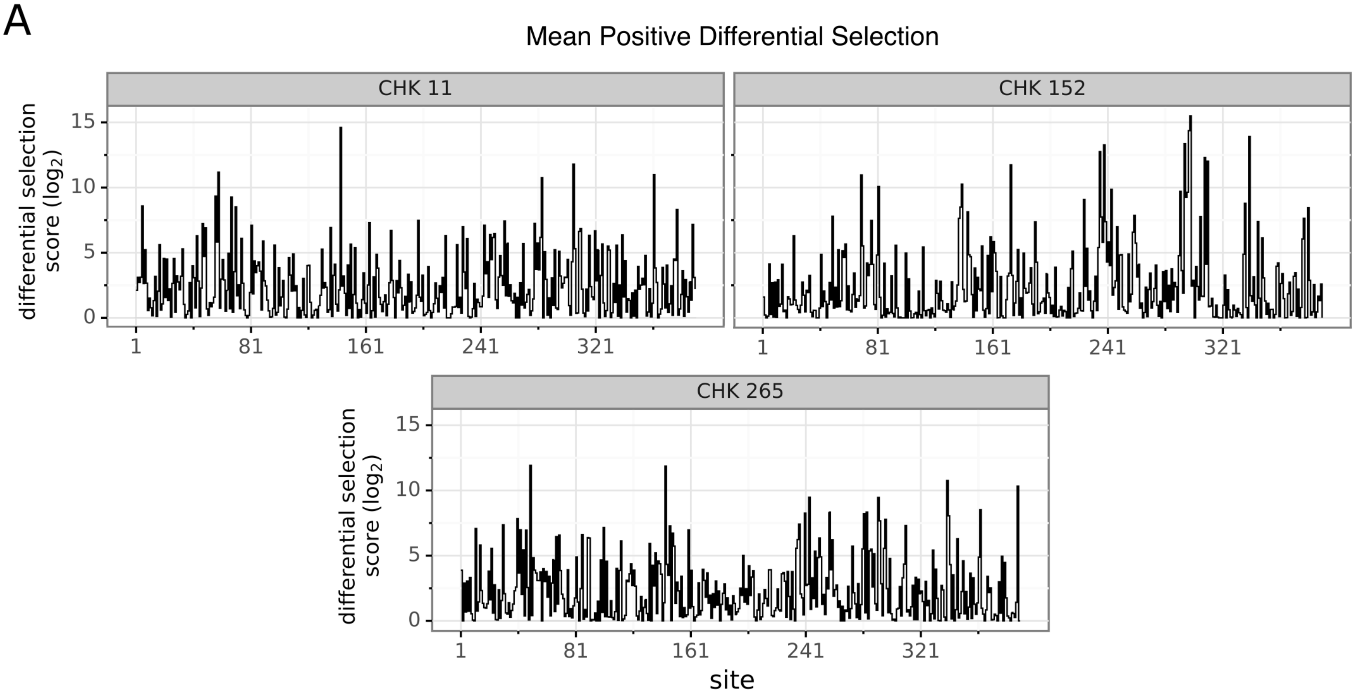

B

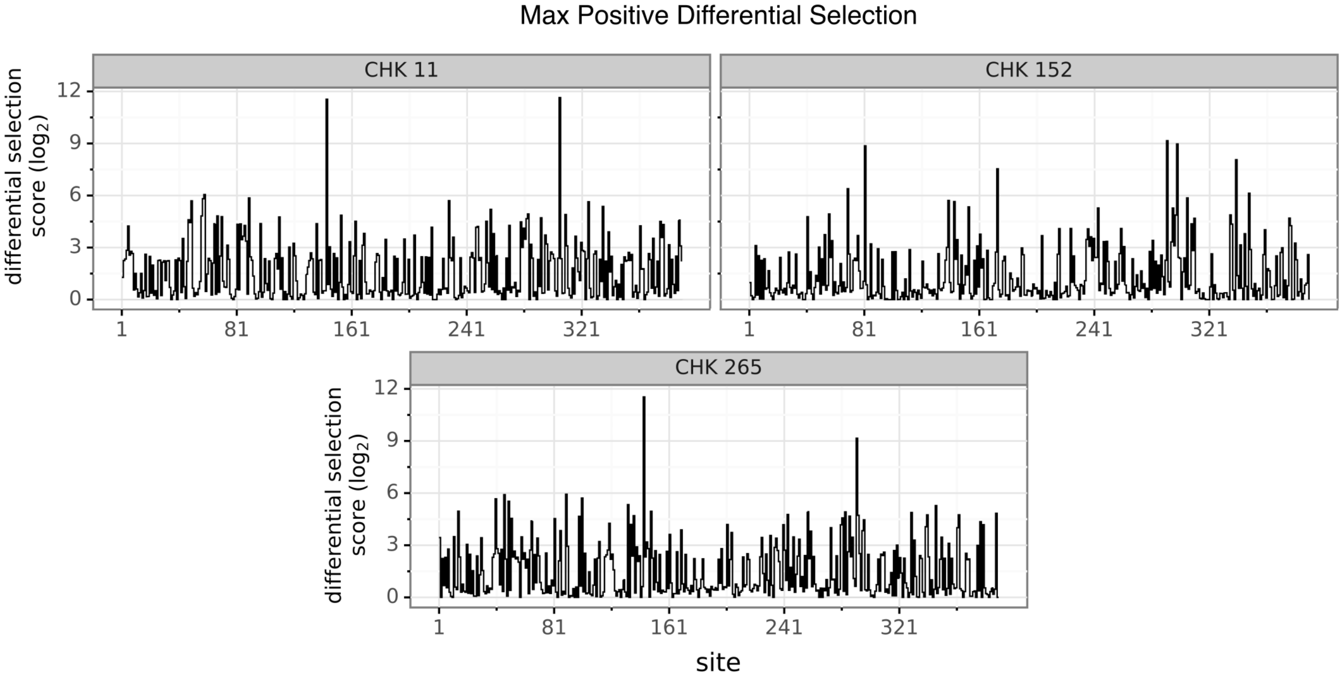

Figure S2. Differential selection line plots for monoclonal antibodies CHK-11, CHK-152, and CHK-265 against the CHIKV-p62-DMS virus library. Using *dms_tools2*, the average differential selection across replicate wells was calculated for each neutralizing antibody against both replicates of virus-only control wells independently and error corrected with the corresponding wtDNA sequencing control. (**A**) For all comparisons, the average positive differential site selection scores are plotted (measured as log_2 scores) for each antibody. (**B**) The highest-scoring positively-selected mutant (measured as log_2 scores) is plotted for each site after averaging across all comparisons for each antibody.

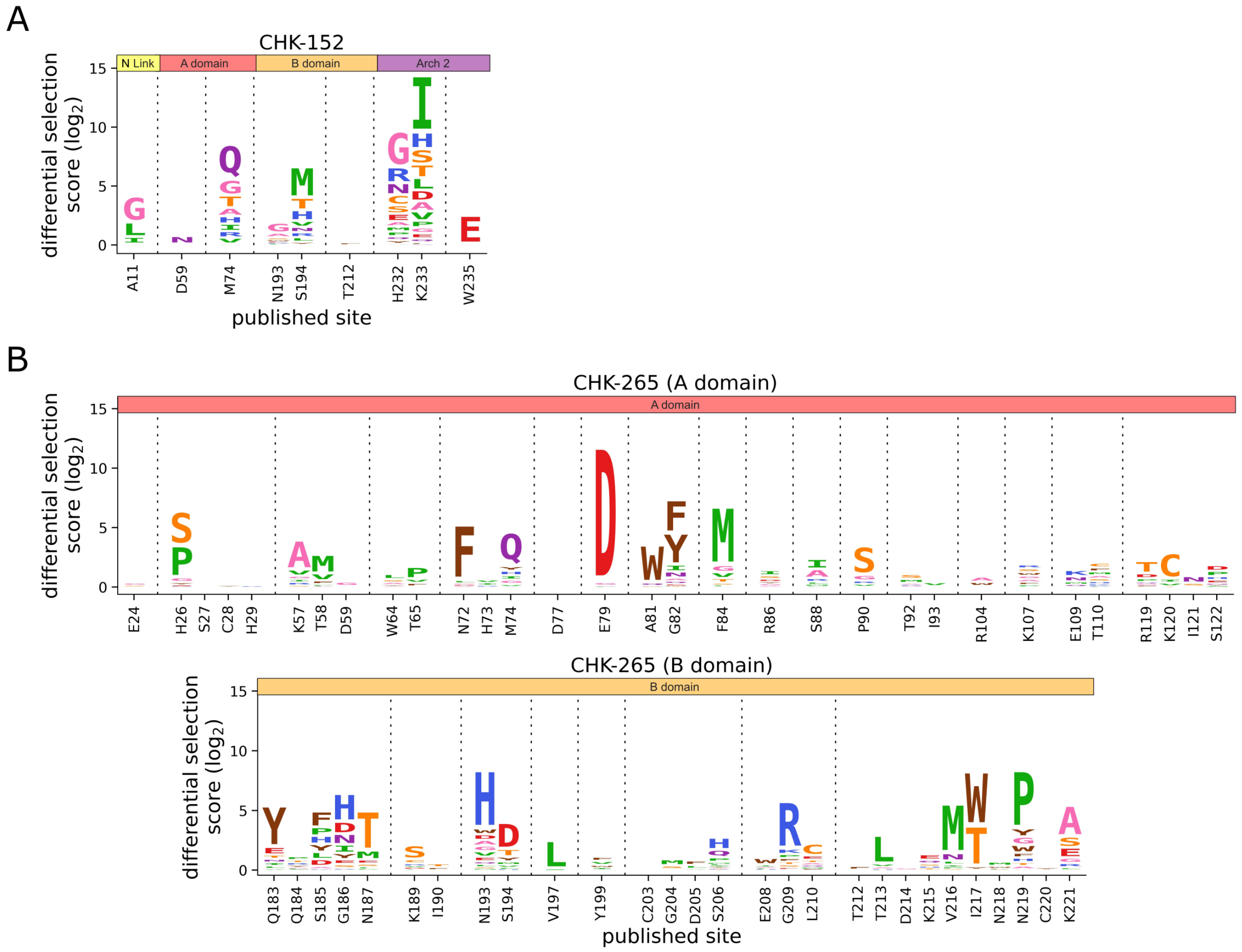

Figure S3. Escape mutations identified at sites previously reported as contacts or critical residues for CHK-152 and CHK-265 monoclonal antibodies. (**A and B**) Previously identified contact sites or other critical residues for antibodies CHK-152 (A) and CHK-265 (B) were recorded (**see Tables S1 and S2**). These sites were extracted from the differential selection dataset following escape mutant selection and positively selected mutants plotted via logoplot. Antibody escape is reported as a $\log_2$ selection score with letter heights corresponding to degree of selection for that mutant averaged across all comparisons with error correction from the wtDNA sequencing control. For (B), due to the large number of important sites identified for CHK-265, the logoplot was split by whether the site resides in the E2 A domain or E2 B domain, respectively.

	Domain	E2 no.	p62 no.	Origin Strain	Origin Residue	Escape Residue	Library Escape Mutants[†]	Residue Mapping Method
**CHK-152**	**A**	11	75	CHIKV-LR	A	NA	G, L, I	Cryo-EM road mapping[1]
		59	123	CHIKV-LR	D	N	**N**	Serial passaging[2]
						NA		Cryo-EM road mapping[1]
		74	138	CHIKV-LR	M	NA	Q, G, T, A, H, I, R, V	Cryo-EM road mapping[1]
	**B**	193	257	CHIKV-LR	N	NA	G, A, S, W, E	Cryo-EM road mapping[1]
		194	258	CHIKV-LR	G	NA	M, T, H, V, N, R, L, Y	Cryo-EM road mapping[1]
		212	276	CHIKV-LR	T	NA	F	Cryo-EM road mapping[1]
		232	296	CHIKV-LR	H	NA	G, R, N, C, S, E, A, M, P, Q, Y	Cryo-EM road mapping[1]
	**β-ribbon connector (Arch 2)**	233	297	rVSV-CHIKV	K	T	I, H, S, **T**, L, D, A, V, P, G, **E**, Q, F	In vivo, neutralization[2,3]
				CHIKV-LR		E		In vivo selection[2]
		235	299	CHIKV-LR	W	NA	E	Cryo-EM road mapping[1]

Table S1. Escape mutations identified at sites previously reported as contacts or critical residues for CHK-152 monoclonal antibody.

	Domain	E2 no.	p62 no.	Origin Strain	Origin Residue	Escape Residue	Library Escape Mutants†	Residue Mapping Method
CHK-265	A	24	88	CHIKV-LR	E	NA	G, S	Cryo-EM road mapping[4]
		26	90	CHIKV-LR	H	NA	S, P, G, Y	Cryo-EM road mapping[4]
		27	91	CHIKV-LR	S	NA	none	Cryo-EM road mapping[4]
		28	92	CHIKV-LR	C	NA	none	Cryo-EM road mapping[4]
		29	93	CHIKV-LR	H	NA	none	Cryo-EM road mapping[4]
		57	121	CHIKV-LR	K	NA	A, V, G, I, R	Cryo-EM road mapping[4]
		58	122	CHIKV-LR	T	NA	M, V, F	Cryo-EM road mapping[4]
		59	123	CHIKV-LR	D	NA	G	Cryo-EM road mapping[4]
		64	128	CHIKV-LR	W	NA	L, S, A, Y	Cryo-EM road mapping[4]
		65	129	CHIKV-LR	T	NA	P, V, F	Cryo-EM road mapping[4]
		72	136	CHIKV-LR	N	NA	F, L, G	Cryo-EM road mapping[4]
		73	137	CHIKV-LR	H	NA	V, I	Cryo-EM road mapping[4]
		74	138	CHIKV-LR	M	NA	Q, Y, H, I, G, V	Cryo-EM road mapping[4]
		77	141	CHIKV-LR	D	NA	none	Cryo-EM road mapping[4]
		79	143	CHIKV-LR	E	NA	D, G	Cryo-EM road mapping[4]
		81	145	CHIKV-LR	A	NA	W, Q	Cryo-EM road mapping[4]
		82	146	CHIKV-LR	G	NA	F, Y, I, N, A, Q, E	Cryo-EM road mapping[4]
		84	148	CHIKV-LR	F	NA	M, G, V, T, S, L	Cryo-EM road mapping[4]
		86	150	CHIKV-LR	R	NA	I, S, D, Y, G, A	Cryo-EM road mapping[4]
		88	152	CHIKV-LR	S	NA	I, A, R, L, M	Cryo-EM road mapping[4]
		90	154	CHIKV-LR	P	NA	S, G, R, V	Cryo-EM road mapping[4]
		92	156	CHIKV-LR	T	NA	S, M, A	Cryo-EM road mapping[4]
		93	157	CHIKV-LR	I	NA	V	Cryo-EM road mapping[4]
		104	168	CHIKV-LR	R	NA	A, W	Cryo-EM road mapping[4]
		107	171	CHIKV-LR	K	NA	R, S, W, G, A, F, P	Cryo-EM road mapping[4]
		109	173	CHIKV-LR	E	NA	K, N, M, Q	Cryo-EM road mapping[4]
		110	174	CHIKV-LR	T	NA	C, F, M, A, S, H, K	Cryo-EM road mapping[4]
		119	183	CHIKV-LR	R	NA	T, D, P, Q, G, F	Cryo-EM road mapping[4]
		120	184	CHIKV-LR	K	NA	C, I, V	Cryo-EM road mapping[4]
		121	185	CHIKV-LR	I	NA	N, S	Cryo-EM road mapping[4]
		122	186	CHIKV-LR	S	NA	D, P, H, E	Cryo-EM road mapping[4]
	B	183	247	CHIKV-37997	Q	NA	Y, E, T, N, I, L	PISA solvent exclusion analysis[5]
		184	248	CHIKV-LR	Q	A	P, T, R, Y	Alanine-scanning mutagenesis[4]
						NA		Cryo-EM road mapping[4]
				CHIKV-37997		NA		PISA solvent exclusion analysis[5]
		185	249	CHIKV-LR	S	A	F, P, H, Y, L, D, **A**	Alanine-scanning mutagenesis[4]
						NA		Cryo-EM road mapping[4]
				CHIKV-37997		NA		PISA solvent exclusion analysis + alanine scanning mutagenesis/neutralization escape[5]
		186	250	CHIKV-37997	G	NA	H, D, N, I, Y, E, S	PISA solvent exclusion analysis + alanine scanning mutagenesis/neutralization escape[5]
		187	251	CHIKV-LR	N	NA	T, M, E, S, L	Cryo-EM road mapping[4]
				CHIKV-37997		NA		PISA solvent exclusion analysis[5]
		189	253	CHIKV-LR	K	NA	S, C, R, V, D	Cryo-EM road mapping[4]
				CHIKV-37997		NA		PISA solvent exclusion analysis[5]
		190	254	CHIKV-LR	I	A	T, F	Alanine-scanning mutagenesis[4]
		193	257	CHIKV-LR	D (N)	NA	H, W, D, A, G, V, E, Q, L, S	Cryo-EM road mapping[4]
		194	258	CHIKV-LR	E (S)	NA	D, T, Y, M, V, L	Cryo-EM road mapping[4]
		197	261	CHIKV-LR	V	A	L, I	Alanine-scanning mutagenesis[4]
		199	263	CHIKV-LR	Y	A	F, V, W, I	Alanine-scanning mutagenesis[4]
		203	267	CHIKV-37997	C	NA	none	PISA solvent exclusion analysis[5]
		204	268	CHIKV-37997	G	NA	M, S	PISA solvent exclusion analysis[5]
		205	269	CHIKV-LR	G (D)	NA	F, L	Cryo-EM road mapping[4]
		206	270	CHIKV-LR	S	NA	H, Q, P, M, L, I, Y	Cryo-EM road mapping[4]
		208	272	CHIKV-LR	E	NA	W, I, A	Cryo-EM road mapping[4]
		209	273	CHIKV-LR	G	A	R, K, P, F, T, S, E	Alanine-scanning mutagenesis[4]
		210	274	CHIKV-LR	L	A	C, E, T, **A**, Q, M	Alanine-scanning mutagenesis[4]
						NA		Cryo-EM road mapping[4]
		212	276	CHIKV-LR	T	A	F	Alanine-scanning mutagenesis[4]
						NA		Cryo-EM road mapping[4]
		213	277	CHIKV-LR	T	NA	L, V, A	Cryo-EM road mapping[4]
		214	278	CHIKV-LR	D	NA	G	Cryo-EM road mapping[4]
		215	279	CHIKV-LR	K	NA	E, Q, S, H, T	Cryo-EM road mapping[4]
		216	280	CHIKV-LR	V	NA	M, N, L, I, P	Cryo-EM road mapping[4]
				CHIKV-37997		NA		PISA solvent exclusion analysis[5]
		217	281	CHIKV-LR	I	A	W, T, Y	Alanine-scanning mutagenesis[4]
		218	282	CHIKV-LR	N	NA	M, W	Cryo-EM road mapping[4]
				CHIKV-37997	N	NA		PISA solvent exclusion analysis[5]
		219	283	CHIKV-LR	N	NA	P, Y, G, W, F, H, T, A	Cryo-EM road mapping[4]
				CHIKV-37997		NA		PISA solvent exclusion analysis[5]
		220	284	CHIKV-37997	C	NA	Y	PISA solvent exclusion analysis[5]
		221	285	CHIKV-37997	K	NA	A, S, E, G, R, L	PISA solvent exclusion analysis[5]

Table S2. Escape mutations identified at sites previously reported as contacts or critical residues for CHK-265 monoclonal antibody.

SUPPLEMENTARY REFERENCES

1. Sun S, Xiang Y, Akahata W, Holdaway H, Pal P, Zhang X, et al. 2013. Structural analyses at pseudo atomic resolution of Chikungunya virus and antibodies show mechanisms of neutralization. *eLife* 2:e00435.

2. Pal P, Dowd KA, Brien JD, Edeling MA, Gorlatov S, Johnson S, et al. 2013. Development of a highly protective combination monoclonal antibody therapy against chikungunya virus. *PLoS Pathog* 9(4):e1003312.

3. Quiroz JA, Malonis RJ, Thackray LB, Cohen CA, Pallesen J, Jangra RK, et al. 2019. Human monoclonal antibodies against chikungunya virus target multiple distinct epitopes in the E1 and E2 glycoproteins. *PLoS Pathog* 15(11):e1008061.

4. Fox JM, Long F, Edeling MA, Lin H, van Duijl-Richter MKS, Fong RH, et al. 2015. Broadly neutralizing alphavirus antibodies bind an epitope on E2 and inhibit entry and egress. *Cell* 163(5):1095–107.

5. Raju S, Adams LJ, Earnest JT, Warfield K, Vang L, Crowe JE, et al. 2023. A chikungunya virus–like particle vaccine induces broadly neutralizing and protective antibodies against alphaviruses in humans. *Sci Transl Med* 15(696):eade8273.
